# Dangers of orbital retraction in transorbital neuroendoscopic surgery

**DOI:** 10.1007/s00701-026-06921-4

**Published:** 2026-06-04

**Authors:** Ayushi Agarwal, Jessica Y. Tong, Dinesh Selva

**Affiliations:** 1 South Australian Institute of Ophthalmology, The Royal Adelaide Hospital, Adelaide, Australia; 2 Sydney Medical School, Faculty of Medicine and Health, The University of Sydney, Sydney, NSW Australia; 3 Discipline of Ophthalmology and Vision Sciences, University of Adelaide, Adelaide, Australia; 4 Faculty of Health and Medical Sciences, The University of Adelaide, Adelaide, South Australia Australia

**Keywords:** Transorbital neuroendoscopic surgery, Orbital retraction, Orbital compartment syndrome, Surgical freedom, Globe retraction

## Abstract

**Purpose:**

This review summarizes the risks associated with orbital retraction in transorbital neuroendoscopic surgery (TONES) and proposes practical strategies to optimize surgical freedom while preserving visual function.

**Methods:**

A qualitative review synthesized through a systematic literature search of PubMed (MEDLINE) database. Studies involving human or cadaveric TONES and endoscopic transorbital approaches were reviewed.

**Results:**

Ten studies met inclusion criteria, comprising cadaveric as well as human subjects. Virtual-reality and cadaveric models revealed that 10–11 mm of globe displacement provides adequate surgical freedom, whereas sustained retraction beyond approximately 15 mm results in steep rises in intraocular and intra-orbital pressure posing a higher risk of orbital compartment syndrome. Clinical reports link prolonged retraction to transient or permanent mydriasis, ocular hypotony, decompression retinopathy, and visual loss.

**Conclusion:**

Orbital retraction is central to TONES but is the principal driver of ocular morbidity. Based on the available evidence, retraction should be dynamic, gentle, and intermittent, with globe displacement generally limited to 10–12 mm. Apex-directed focal pressure should be avoided, and continuous monitoring of pupil and intraocular pressure is essential. Multidisciplinary protocols are recommended to standardize safe retraction practices while preserving the advantages of TONES.

## Introduction

Transorbital neuroendoscopic surgery (TONES) has garnered significant attention as an innovative approach to access the skull base and cranial fossa via minimally invasive surgical corridors. In 2010, Moe et al. described utilization of the orbit as a working channel, where the endoscope provided a coplanar trajectory to the anterior cranial fossa (ACF) and middle cranial fossa (MCF) [34]. In order to achieve adequate surgical freedom, the globe and orbital soft tissues must be adequately retracted for deeper access.

The utility of TONES has now expanded to include the posterior cranial fossa (PCF) with several advantages, such as avoidance of a craniotomy, minimized brain retraction, reduced surgical morbidity, and quicker post-operative rehabilitation [8, 19, 31, 41].

Despite these expanding indications, the current literature predominantly focuses on surgical access, technique, and neurosurgical success rates. There remains a significant gap in knowledge regarding ophthalmic morbidity associated with this approach, particularly the nuanced threshold between adequate surgical freedom and critical ischemic or compressive injury to the orbit. Therefore, the objective of this review is to bridge the gap by systematically synthesizing the available evidence on the complications of orbital retraction. By providing an orbital surgeon's perspective, this study uniquely aims to establish evidence-based parameters for safe retraction and propose practical, multidisciplinary strategies to mitigate iatrogenic visual loss.

## Methodology

A systematic literature review relevant to TONES was conducted through the PubMed database. Studies identifying orbital or visual complications, hemodynamic or intraocular pressure-related effects, pupillary, and motility changes associated with orbital retraction in TONES, were identified. Reference lists of included articles and prior reviews on TONES and orbital surgery were manually screened to identify additional relevant studies.

Studies were eligible if they involved either human or cadaveric models of TONES or other endoscopic transorbital approach. Given the limited number of studies directly quantifying orbital retraction in TONES, eligible studies included those that either, a) directly measured or described the extent and technique of orbital retraction, or b) provided mechanistic, anatomic, or clinical evidence on orbital and ocular complications, pupillary dynamics, intraocular and intra-orbital pressure changes, or visual outcomes pertinent to orbital retraction during endoscopic transorbital approaches. Conference abstracts, non-English language articles, reports on TONES unrelated to orbital retraction, reviews, and articles with non-transorbital cranial approaches, were excluded.

**Fig. 1:**
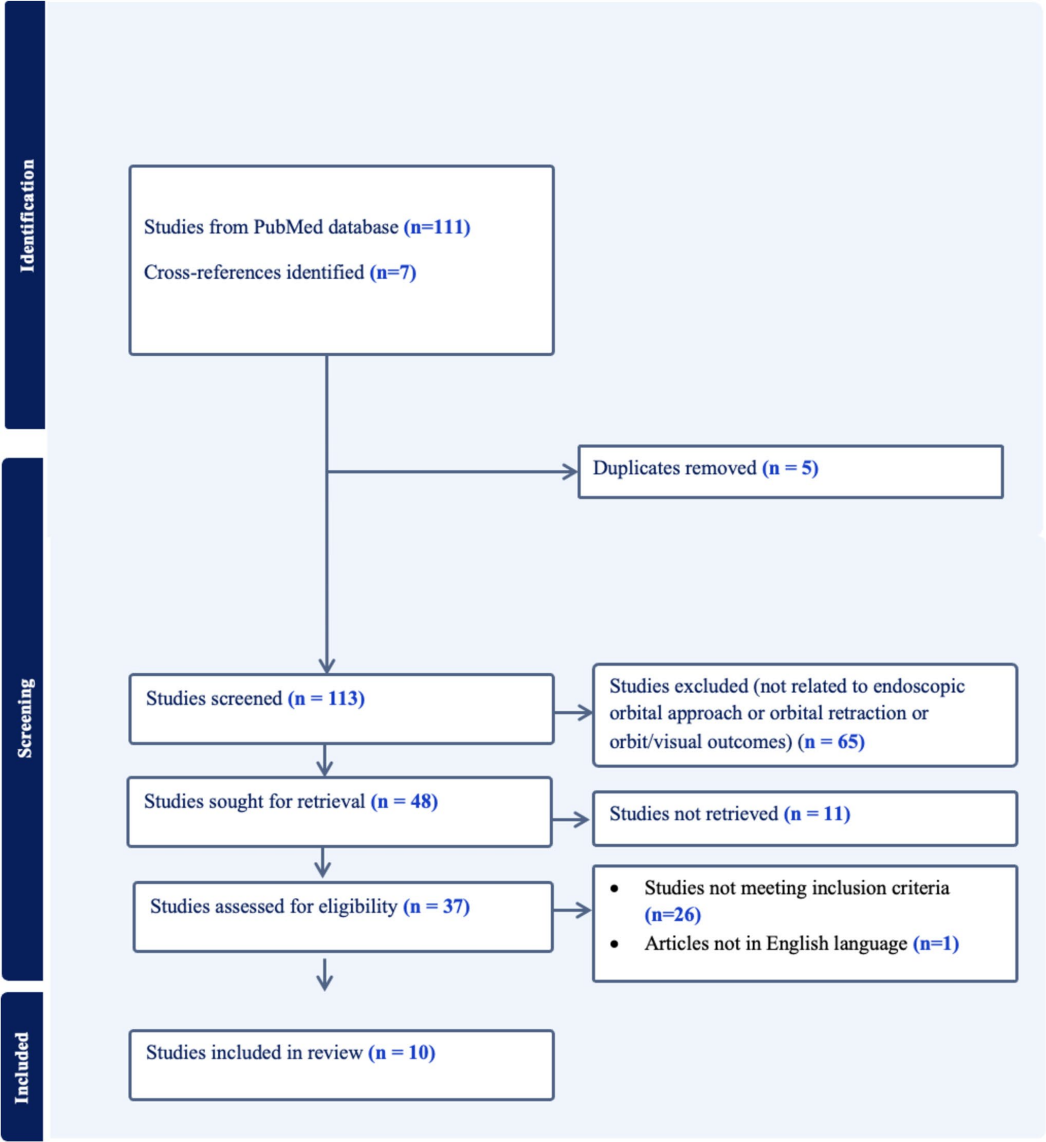
PRISMA flow diagram depicting study selection for the qualitative review evaluating orbital retraction in TONES

This review was informed by the Preferred Reporting Items for Systematic reviews and Meta-analyses (PRISMA) guidelines (Fig. 1). However, due to the significant methodological heterogeneity of the included literature, which predominantly comprised cadaveric studies, virtual-reality models, and isolated retrospective clinical case reports, a formal quantitative meta-analysis and robust statistical evaluation were precluded. Two reviewers independently screened titles and abstracts for relevance (AA, JT), followed by full-text assessment of potentially eligible studies. Any disagreements were resolved by a third reviewer (DS).

To contextualize the TONES-specific literature, key studies on orbital compartment syndrome, ocular hypotony, decompression retinopathy, pupillary dynamics and visual loss after orbital surgery were also reviewed and integrated into the discussion if they provided insights applicable to TONES. Consequently, a qualitative synthesis was performed, acknowledging the inherent selection and publication biases associated with this review.

The search strategy included combination of Medical Subject Headings (MeSH) and free-text terms using Boolean operators (AND/OR) as follows: (“transorbital neuroendoscopic surgery” OR “endoscopic transorbital” OR “transorbital approach” OR “transpalpebral” OR “transorbital endoscopic”) AND (“orbital retraction” OR “globe retraction” OR “orbital compartment syndrome” OR “intraocular pressure” OR “orbital pressure” OR “orbital surgery” OR “vision loss” OR “central retinal artery occlusion” OR “mydriasis” OR “ocular hypotony” OR “orbital manometry” OR “cranial nerve palsy”).

## Results

A total of 118 articles were identified in the initial search, with 7 additional pertinent cross-references. Following title and abstract screening, 37 full-text articles were assessed for eligibility. Ultimately, 10 studies met the inclusion criteria and were included in the qualitative synthesis (Table 1). The extracted data from 3 quantitative studies and 7 qualitative studies revealed a consistent, critical threshold of 10-12mm of globe retraction with maximal surgical freedom while maintaining safe limits, whereas sustained retraction exceeding 15 mm strongly correlated with abrupt, exponential increases in IOP and IORP, significantly heightening the risk of ischemic and compressive visual complications.

**Table 1:** Summary of the included studies providing quantitative or qualitative evidence

S. No	Author/year	Study design	Parameters assessed	Outcomes & conclusion	Nature of evidence
1	Kim et al./2021 [27]	5 cadavers + 9 clinical cases	IOP and IORP	Curvilinear rise in IOP/IORP; critical inflection > 15 mm of globe retraction. IORP satisfactorily reflected the IOP	Quantitative
2	Piper et al./2024 [39]	Virtual-reality model	Degree of freedom, area of freedom by retraction	Optimal retraction window of 10–11 mm with 15 mm as conservative limit	Quantitative
3	Tong JY et al./2025 [47]	Cadaveric study	Working corridor dimensions	Within a fixed 10 mm retraction ceiling, exposure expanded linearly with soft tissue/bone release. This study demonstrates that corridor depth is gained by graded landmark release, rather than greater displacement	Quantitative
4	Ramakrishna et al./2016 [41]	40 clinical cases	Visual and ophthalmic complications	No visual complications. Attributes minimal morbidity to limiting orbital retraction to < 10 mm, with intermittent instrument removal every 15–20 min	Qualitative
5	Austria et al./2026 [1]	35 clinical cases	Visual and ophthalmic complications	Multi-disciplinary settings warranted for minimal visual morbidity. Nil ophthalmic complications	Qualitative
6	Foulsham et al./2022 [12]	Case of spheno-orbital meningioma	Visual and ophthalmic complications	Multilayered macular haemorrhage post-TONES; vision 20/400 with no mydriasis	Qualitative
7	Kong et al./2019 [29]	18 clinical cases	Complications (ophthalmic and non-ophthalmic)	Drilling the greater wing of the sphenoid in lateral wall negates the need for excessive globe retraction, leading to reduced surgical morbidity	Qualitative
8	Dallan et al./2018 [7]	14 clinical cases	Complications, proptosis reduction, reduction in tumor volume	Demonstrates that with subperiosteal dissection and optimal retraction (no quantified displacement reported), the superior eyelid approach can be performed without new visual loss or worsening diplopia	Qualitative
9	Houlihan et al./2024 [19]	Cadaveric study	Degree of surgical freedom	Biportal transorbital approach did not offer superior SF. The study reinforced that SF in TONES is constrained by orbital geometry and globe displacement, favouring adjunctive orbitotomy over escalating retraction	Qualitative
10	Lin BJ et al./2019 [31]	Cadaveric study	Bony landmarks, working area, and exposure axes	Defines inferolateral transorbital corridor and superior orbital retraction technique required to access IOF; supports that bony widening (lateral rim osteotomy, IOF expansion) substitutes for aggressive globe displacement	Qualitative

*IOP* intraocular pressure, *IORP* Intraorbital pressure, *CRAO* Central retinal artery occlusion, *TONES* Transorbital neuroendoscopic surgery, *SF* Surgical freedom, *IOF* Inferior orbital fissure

## Discussion

### Principles and concepts of orbital retraction

An ideal surgical corridor is conceptually characterized by the triad of maximum instrument maneuverability, termed as surgical freedom (SF), optimal visualization, and a focus on the shortest, coplanar trajectory to the area of interest [20]. In a recent analysis by Piper et al., virtual-reality models of transpalpebral orbital rim-preserving endoscopic orbitotomy (TORPEDO) were studied with the anterior clinoid process (ACP) as the surgical target to measure the degree and area of surgical freedom achieved with medial globe retraction. The study revealed that the maximal surgical corridor required at least 10–11 mm of medial globe retraction for ease of maneuverability and visualization, with an upper limit of 15 mm, beyond which significant globe compression against the bony orbit ensues [39].

The authors reiterate three-pronged approach for orbital retraction. Firstly, retraction should be dynamic and intermittent rather than rigid and prolonged. Secondly, retraction should be minimized where necessary with the safe range being 10–15 mm. Thirdly, in TONES, the subperiosteal plane is ideal for circumventing direct globe compression so that focal, apex-directed pressure on the optic nerve and ophthalmic neurovasculature can be avoided.

### Risks and effects of orbital retraction

#### Effect on pupil

Vigilant intra-operative assessment of pupillary dynamics is imperative during TONES. Pupillary size as well as the configuration can provide vital real-time feedback of any potential iatrogenic visual compromise. Mydriasis is usually a more reliable indicator of neuronal and vascular injury than a relative afferent pupillary defect (RAPD), which may go undetected intraoperatively [35].

**Fig. 2:**
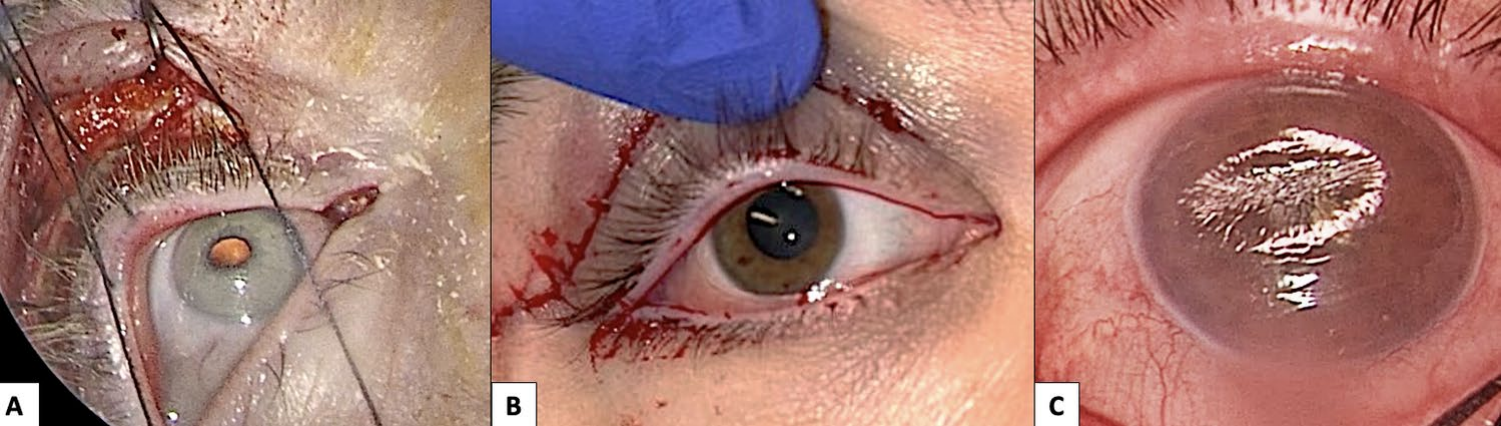
**A**) Intra-operative photograph showing transient oval mydriasis, which resolved on withdrawal of retraction with no associated visual loss. **B**) Fixed, circular, dilated pupil associated with visual loss. **C**) Descemet membrane folds with shallow anterior segment and soft globe, suggestive of intra-operative globe hypotony

The shape, and pattern of mydriasis correlate with surgical parameters such as the approach and duration of orbital retraction. With the superior eyelid crease incision becoming increasingly popular to gain access into the ACF and MCF [17], prolonged retraction in the superolateral orbit can lead to ciliary ganglion damage, owing to its anatomic location being deep and lateral to the optic nerve within the muscle cone [43, 47]. An oval pattern of mydriasis usually occurs due to damage of post-ganglionic, parasympathetic, myelinated short ciliary nerves, which is seen more commonly in surgeries involving the anterior two-thirds of the orbit (Fig. 2A) [35]. Prompt recognition and withdrawal of retraction and instrumentation may restore the pupil size, indicating transient hypoperfusion of ciliary ganglion (CG).

Prolonged orbital retraction or focal posterior retraction affecting vital structures such as the optic nerve (ON), may result in irreversible ischemia of the CG, optic nerve or retina, secondary to prolonged hypoperfusion of the pial vessels posteriorly and central retinal artery anteriorly. This leads to a fixed, circular, and dilated pupil, which may be indicative of permanent visual loss secondary to a central retinal artery occlusion (Fig. 2B) [22, 35].

Although mydriasis may not invariably coincide with visual loss and vice versa, any amount of intraoperative mydriasis, particularly circular, must be managed promptly. In the event of persistent circular mydriasis that does not resolve despite prompt withdrawal of instrumentation, shifting to an alternate surgical approach or amending surgical goals of resection, may be more appropriate [35]. A trial of intravenous steroids can be considered, but has been shown to be of little to no benefit in restoration of associated visual loss [21]. Notably, no instance of intra-operative mydriasis in conjunction with vision loss during TONES has been reported in the literature [30].

#### Effect on intra-ocular pressure (IOP) and intra-orbital pressure (IORP)

In the general population, the normal range of intra-ocular pressure (IOP) and intra-orbital pressure (IORP) varies between 7–21 mmHg and 3–6 mmHg, respectively [28, 42]. These baseline values are higher in certain conditions, such as thyroid eye disease (TED), with a resting orbital tension of 9.7 ± 4.8 mmHg and significantly reduced orbital compliance compared to normal, healthy orbits [27].

External compression, as would be experienced during globe retraction in TONES, is known to alter IOP as well as IORP [27]. Kim et al. analyzed the effects of graded globe retraction in both cadaveric and clinical settings, and found a curvilinear increase of IOP and IORP. Additionally, a strong correlation between IOP and IORP was observed, with the Pearson correlation coefficient of 0.824. Through in vivo continuous pressure monitoring, this study demonstrated that while the pressures rose in a gradual fashion till 15 mm of retraction, a sudden and significant spike was noted when the medial globe was retracted beyond 15 mm to 20 mm [27].

This sharp rise in the IORP within the tight confines of the orbit can lead to an orbital compartment syndrome (OCS), which is a blinding ophthalmic emergency. The acute spike in IOP causes direct or ischemic damage to the optic nerve, which if prolonged, results in permanent visual loss. Studies have shown IOP as the most reliable indicator for OCS, with urgent surgical intervention warranted if the IOP exceeds 30–40 mmHg [4, 36]. Hayreh and Weingeist, in their animal model study, advocated for immediate pressure release and decompression within 98–105 min of onset, in order to reverse acute ischemic injury [18].

When critically comparing these findings across the literature, a distinct difference emerges between experimental models and clinical reality. While virtual-reality and cadaveric studies precisely define the 15 mm retraction threshold for pressure spikes, these models cannot fully replicate in vivo vascular compliance, real-time hemodynamic fluctuations, or pre-existing patient comorbidities (e.g., rigid orbits in thyroid eye disease). Consequently, the actual clinical threshold for ischemic injury may be lower than what experimental models suggest. This discrepancy underscores the necessity for continuous intraoperative pupil and pressure monitoring in clinical practice, rather than relying solely on fixed, model-derived retraction distances.

#### Ocular hypotony 

On the other end of the spectrum is hypotony, defined as an IOP of less than 6.5 mmHg. Ocular hypotony should prompt consideration for an occult globe perforation or rupture, but it has also been linked to surgical manipulation and globe compression during orbital fracture repair or biopsy, where it is often self-limiting [15, 44]. Schein et al. functionally equated intraoperative globe compression to ocular massage, which results in reduction of aqueous inflow, secondary to neurovascular dysfunction, ciliary body edema, and circulatory changes [40]. Gowda et al. hypothesized that intraoperative ciliary body hypoperfusion with resultant edema may be the plausible mechanism responsible for hypotony [15].

Intraoperatively, ocular hypotony is identifiable by distinct signs of a soft globe, corneal Descemet membrane folds, and a shallow anterior segment (Fig. 2C). Although the majority of cases resolve with cessation of inciting surgical factors, in persistent cases of hypotony, vision threatening sequelae, such as ciliochoroidal effusion, hypotonous maculopathy, choroidal detachment, iridocorneal touch due to complete collapse, may ensue [49, 52].

Earlier work on the IOP-lowering effect of consistent mild external compression versus intermittent digital massage has revealed higher reduction in IOP with the former method [11, 45]. Extrapolating these findings to TONES, persistent globe compression due to prolonged retraction is likely to exert a greater risk of hypotony than intermittent retraction. Sudden hypotony upon release of retraction may also predispose to serous or hemorrhagic choroidal detachment due to a rise in the transmural pressure across choroidal vessels. One should also be cautious in patients with thin sclera from high myopia or autoimmune conditions, where there is a lower threshold for these pressure differentials induced by retraction [25].

#### Visual loss

The incidence of vision loss from orbital surgery particularly at the apex, carries an overall incidence varying from 0.44 to 0.84%, with a higher risk reported with a combination of craniotomy and orbitotomy (18%) and where bony optic canal is surgically intervened due to direct optic nerve manipulation [3, 21]. Several studies have consistently shown that the apical location of orbital tumors, presence of tight intraoperative adhesions, and severe displacement of the ON, to be significant predictors of poor visual prognosis following surgery for orbital tumors [23, 50].

While there have been no reported cases of vision loss directly attributed to TONES procedure, there is one case of no light perception in a sphenorbital meningioma following a pterional transpalpebral approach. The authors reported a preoperative acuity of counting fingers, and attributed the postoperative visual loss to tumor invasion into the orbit and cavernous sinus, rather than an iatrogenic event [53]. Foulsham et al. described a case of a spheno-orbital meningioma, which underwent an uneventful transorbital endoscopic resection, and was complicated in the immediate post-operative period by the development of a multi-layered intraocular hemorrhage with deterioration of visual acuity to 20/400 from 20/20. Interestingly, the intracranial pressure (ICP) remained normal and no intraoperative mydriasis or hemodynamic changes were observed [12]. The authors hypothesized that globe or ON manipulation during surgery may have led to a rise in venous pressure, leading to retinal capillary rupture. Prolonged and improper globe retraction techniques can either lead to indirect, ischemic damage of the optic nerve and the retina, or a direct injury to the vital structures such as stretch or compression of the optic nerve with compromised visual acuity, the latter of which is rarer [37, 46].

##### Direct mechanism

Direct injury to the optic nerve (ON) through traction or compression is uncommon, although the risk should be considered in TONES if the working field is in close proximity to the nerve.

Prolonged surgery and inadvertent mechanical trauma from orbital retractors are significant factors for the development of corneal abrasion and epithelial defect during orbital surgery, resulting in diminished visual acuity [16]. When retracting the orbit, care must be taken to safeguard the contralateral globe as well. Specific measures include the use of lubricating ointment with taping of the contralateral eye and for the operative eye, a corneal cover with lubricating ointment, or occasionally, a temporary tarsorrhaphy [24].

##### Indirect mechanism

Knowledge of the ocular perfusion pressure (OPP) is useful to understand the vascular mechanism responsible for visual loss. OPP is the difference between mean arterial pressure and intraocular pressure, and shares an inverse relationship with IOP [5]. Raised IOP can acutely impact retinal perfusion as well as inner retinal function and thereby cause OCS. If IOP exceeds the mean arterial pressure in the central retinal artery (CRA) and short posterior ciliary artery, retinal and ON perfusion becomes compromised [13]. Prolonged vascular compromise results in ischemic optic neuropathy, central retinal artery occlusion, and ophthalmic artery occlusion [9]. Direct vasospasm of the ophthalmic artery branches can further precipitate ischemia. Although, calcium channel antagonists such as nimodipine, are widely used as rescue therapy for cerebral vasospasm, it is unclear if they have an equivalent benefit for the ophthalmic circulation [51]. Pressure fluctuations with sudden decompression of the orbit during surgery can also lead to visual compromise, owing to sudden rise of transmural pressure across choroidal vessels resulting in choroidal effusion, optic disc edema, hypotonous maculopathy, and ciliochoroidal detachment. This is similar to the pathogenetic mechanism underlying intraocular hemorrhage secondary to prolonged and severe scleral indentation followed by sudden release during retinopathy of prematurity examination [32].

#### Miscellaneous

While performing TONES, aggressive globe retraction can inflict inadvertent trauma to the recti muscles, especially lateral rectus, leading to edema, paresis, and consequent diplopia [29]. Facial paresthesia is another reported complication, likely attributable to manipulation or injury to the branches of the trigeminal nerve (V1) or supraorbital nerve bundle during surgery, which is usually self-limiting [1, 7]. Ptosis is a common postoperative finding following a transpalpebral approach, but has been found to gradually resolve over a period of several months [48]. It is thought to occur due to tractional injury to the superior rectus-levator muscle complex [29].

### Practical recommendations

As orbital surgeons, the authors recommend adoption of certain strategies to minimize risks associated with globe retraction in TONES.

#### Pre-operative considerations

Cases should be evaluated for prior corneal refractive surgeries, particularly radial keratotomy (RK), high myopia, scleromalacia, and conditions associated with thin sclera such as connective tissue disorders, as these globes may be more susceptible to rupture from prolonged globe compression or manipulation [33, 38]. Additional risk in eyes with pathological myopia is the tendency for causing retinal detachment [10]. Globes with altered IOP such as glaucoma or operated trabeculectomy are likely to be more sensitive to intraoperative pressure fluctuations. Patients with optic neuropathy may be at greater risk of ischemic damage due to pre-existing compromised blood flow in the pial plexus.

#### Intraoperative considerations

- The authors recommend practising dynamic, gentle and intermittent retraction. Orbital retraction should be applied in short cycles, measured in minutes rather than as a continuous state throughout the procedure. The intermittent release of retraction should be gentle in order to prevent decompression retinopathy, which occurs due to sudden IOP fluctuation [2]. Focal pressure at the apical structures should be avoided. Orbital retraction should be minimized, with an average displacement of 10–11 mm and should not exceed 15 mm. In cases where adequate visualization is not achieved with 15 mm of retraction, adjunctive orbitotomy procedures should be considered to expand the working corridor.
- Intraoperatively, a corneal shield may be applied intermittently and the pupil monitored, with adequate lubrication of the cornea with viscoelastic agents [9]. Additionally, the pupil should not be pharmacologically dilated prior to surgery.
- Aggressive deliberate hypotension should be avoided intraoperatively. Although concrete evidence regarding the risks of inducing intra-operative hypotension is lacking, there is a potential risk of hypoperfusion to vital structures, which may be exacerbated by orbital retraction [6].
- In the event that ocular hypotony occurs, the operating surgeon should explore for an occult perforation. Persistent post-operative hypotony due to ciliary body hypoperfusion may benefit from postoperative topical cycloplegics and corticosteroid eye drops [26].

#### Post-operative considerations

Serial monitoring of visual acuity and pupillary status is essential following TONES, especially in the initial 24–48 hours, where the risk of complications, such as retrobulbar hemorrhage is the highest [14]. Signs of orbital compartment syndrome should alert the team for immediate intervention. Although usually transient in nature, complications such as ptosis, V1 paraesthesia, and limited motility should be periodically monitored.

## Limitations

This review is subject to several limitations. First, the existing literature on TONES-related orbital complications is relatively sparse and heavily reliant on cadaveric models, virtual-reality simulations, and retrospective clinical case reports or small series. This precludes a robust statistical analysis and introduces potential publication bias, as uncomplicated cases or minor transient complications may be systematically underreported. Furthermore, there is considerable heterogeneity in the surgical techniques, duration of retraction, and measurement tools used across the included studies. Future large-scale, prospective, multidisciplinary registries are needed to systematically validate these retraction thresholds and standardize intraoperative monitoring protocols.

## Conclusion

The advent of TONES represents a paradigm shift in skull base and transcranial surgery, with the orbit as an ideal surgical corridor. However, the promise is tempered by the risk of ocular morbidity, primarily linked to intraoperative orbital retraction. By using dynamic, gentle, and intermittent retraction; avoiding apex-directed force; coordinating with anaesthesia to preserve ocular perfusion; and embedding pupil and pressure monitoring into the operative routine, neurosurgeons can mitigate potential visual complications. The authors encourage safe retraction practices and promote collaborative protocols between neurosurgeons, ophthalmologists, and anesthetists to preserve visual function while maintaining the advantages of TONES.

## Data Availability

No datasets were generated or analysed during the current study.
